# High surface area nitrogen-functionalized Ni nanozymes for efficient peroxidase-like catalytic activity

**DOI:** 10.1371/journal.pone.0257777

**Published:** 2021-10-12

**Authors:** Anuja Tripathi, Kenneth D. Harris, Anastasia L. Elias

**Affiliations:** 1 National Research Council Canada, Nanotechnology Research Centre, Edmonton, Edmonton, Canada; 2 Department of Chemical and Materials Engineering, University of Alberta, Edmonton, Canada; 3 Department of Mechanical Engineering, University of Alberta, Edmonton, Canada; Indian Institute of Technology Kharagpur, INDIA

## Abstract

Nitrogen-functionalization is an effective means of improving the catalytic performances of nanozymes. In the present work, plasma-assisted nitrogen modification of nanocolumnar Ni GLAD films was performed using an ammonia plasma, resulting in an improvement in the peroxidase-like catalytic performance of the porous, nanostructured Ni films. The plasma-treated nanozymes were characterized by TEM, SEM, XRD, and XPS, revealing a nitrogen-rich surface composition. Increased surface wettability was observed after ammonia plasma treatment, and the resulting nitrogen-functionalized Ni GLAD films presented dramatically enhanced peroxidase-like catalytic activity. The optimal time for plasma treatment was determined to be 120 s; when used to catalyze the oxidation of the colorimetric substrate TMB in the presence of H_2_O_2_, Ni films subjected to 120 s of plasma treatment yielded a much higher maximum reaction velocity (3.7⊆10^−8^ M/s vs. 2.3⊆10^−8^ M/s) and lower Michaelis-Menten coefficient (0.17 mM vs. 0.23 mM) than pristine Ni films with the same morphology. Additionally, we demonstrate the application of the nanozyme in a gravity-driven, continuous catalytic reaction device. Such a controllable plasma treatment strategy may open a new door toward surface-functionalized nanozymes with improved catalytic performance and potential applications in flow-driven point-of-care devices.

## Introduction

Enzymes are complex biological structures that play key roles in metabolic activities and catalyze numerous biological reactions with excellent catalytic activity, efficiency, and selectivity. These natural enzymes, however, also generally require well-controlled reaction conditions (temperature, pH, purity, etc.) [1], and outside of the human body, precisely controlling these operation and storage conditions can be very difficult, which limits commercial applications [2]. As an alternative, artificial enzymes known as “nanozymes” are nanomaterials with enzyme-like characteristics [3, 4]. Nanozymes have attracted enormous research interest in recent years for their unique advantages (low cost, tunable catalytic activity, stability under a wide range of conditions, and ease of mass production), which make them candidates for applications in biosensing, tissue engineering, therapeutics, and environmental protection [5, 6].

Peroxidase is an important natural enzyme that assists in a wide range of physiological reaction pathways [7, 8]. Since the first evidence of ferromagnetic peroxidase mimetics was reported in 2007 [9], various nanomaterials have been identified that possess intrinsic peroxidase-like activity. These include graphene oxide [10], gold nanoparticles [11], and metal-organic frameworks (MOFs) [12]. One reason that nanomaterials are effective as nanozymes stems from their large specific surface area, which results in both a high surface energy and concentration of catalytically active sites [13]. Surface energy can often be further enhanced by surface modification, utilizing methods such as the introduction of surfactants, or doping of various atoms such as phosphorus, sulfur, and nitrogen [14, 15]. For instance, N-doped MoS_2_ demonstrated higher peroxidase-like catalytic activity than undoped MoS_2_ [14]. Similarly, hydrothermal doping of MoO_2_ nanobelts with N and S led to efficient electrocatalytic activity in a hydrogen evolution reaction [16]. In each case, the improved performance was attributed to improved charge transport as a result of increased electron density. Several challenges, however, remain in the implementation of both peroxidase-like nanozymes and other types of nanozymes. First, most of the reported nanozymes are nanomaterials dispersed in solution, rendering the materials hard to recover and reuse. Second, nanozymes tend to have lower densities of active sites by mass than natural enzymes, resulting in reduced enzyme-like activity [17, 18]. Solving either of these challenges would lead to more practical nanozyme materials.

**Fig 1:**
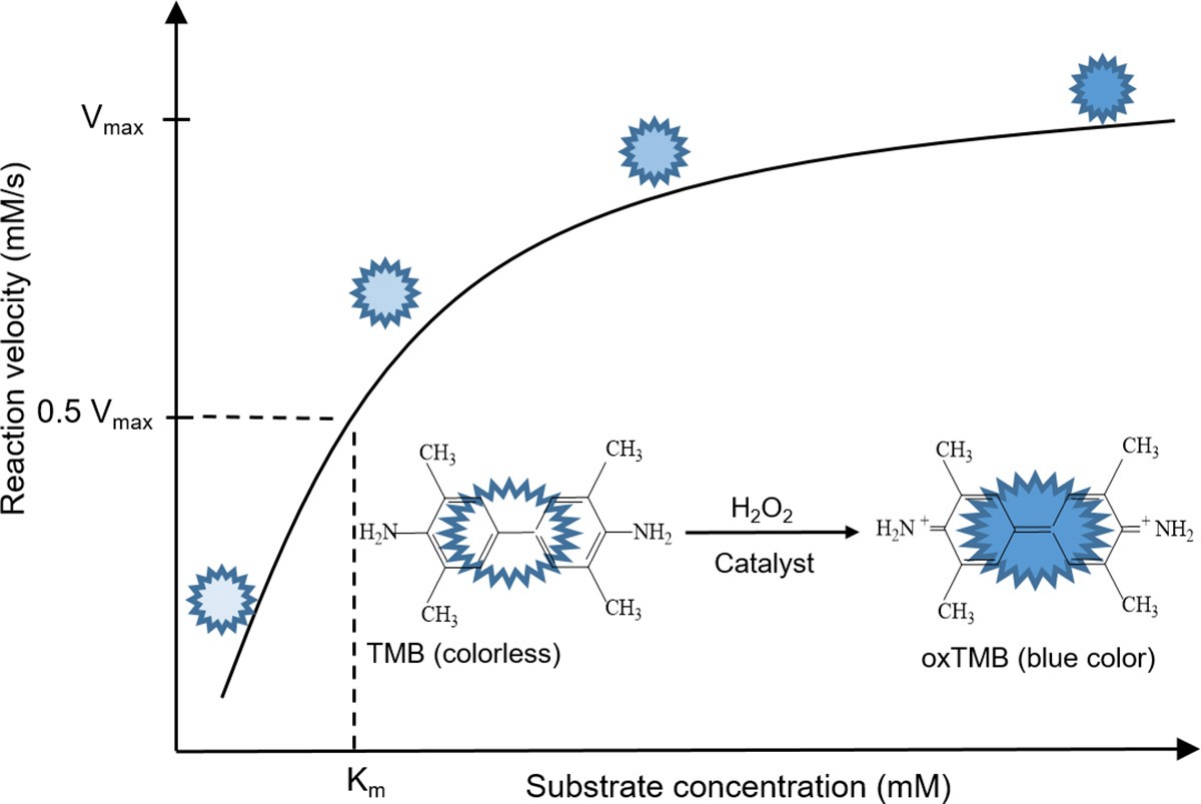
Kinetic curve for peroxidase-like reaction using TMB as a substrate.

Numerous research groups (including our own) have examined the use of high-surface area thin films deposited by glancing angle deposition (GLAD) as catalytic materials [13, 19, 20]. GLAD is a physical vapor deposition technique that exploits atomic shadowing and dynamic motion control to engineer nanostructures with high surface area and controlled porosity [21]. During GLAD, vapor flux is incident upon the substrate at glancing angles (>70° with respect to the surface normal). The porous nanosized columnar thin films fabricated by GLAD are separated by wide gaps and possess high internal porosity [22]. By employing dynamic substrate motion, the GLAD technique also enables the fabrication of modified nanostructures with different shapes such as vertical columns, zigzags, helices, and slanted posts [20, 23]. As they possess an enormous internal surface area that is easily accessible to ambient species, these GLAD-based structures have previously been employed as biosensors for various substrates [21, 23, 24]. In our own previous work, we demonstrated the peroxidase-like behavior of helically-structured Ni GLAD films using TMB as a substrate. This process is illustrated in Fig 1, where the oxidation of colorless TMB (the substrate) into blue oxTMB (the product) occurs in the presence of H_2_O_2_ and the peroxidase-like catalyst [13]. In this previous work, the oxTMB product was further used as an optical sensor for uric acid (UA).

In the present work, we aim to improve the GLAD-based nanozymes’ peroxidase-like activity through nitridation of these nanostructured films. Building on the above-mentioned work on surface modification, we deposit nanocolumnar Ni GLAD films and then N-functionalize these films in an NH_3_ plasma. In a plasma treatment, energetic species bombard the target surface, causing topographical changes and incorporating various surface functional groups [25]. We utilize the plasma-modified and pristine Ni films to catalyze the oxidation of TMB by H_2_O_2_, and we show that the N-functionalized films present enhanced catalytic reaction rates. We demonstrate significantly improved catalytic performance parameters that are among the best in the literature for monometallic, surface-functionalized nanozymes. To further demonstrate the utility of the Ni nanozyme, we also assemble a simple gravity-driven continuous reaction device capable of converting TMB to oxTMB (as shown in Fig 1). The device layout demonstrates a simple GLAD-based thin film architecture that leads toward integration into portable, point of care devices for disease diagnostics. This device structure represents a step toward the introduction of nanozyme structures in real-world applications.

## Results and discussion

### Fabrication and characterization of pristine and nitrogen-functionalized Ni films

**Fig 2:**
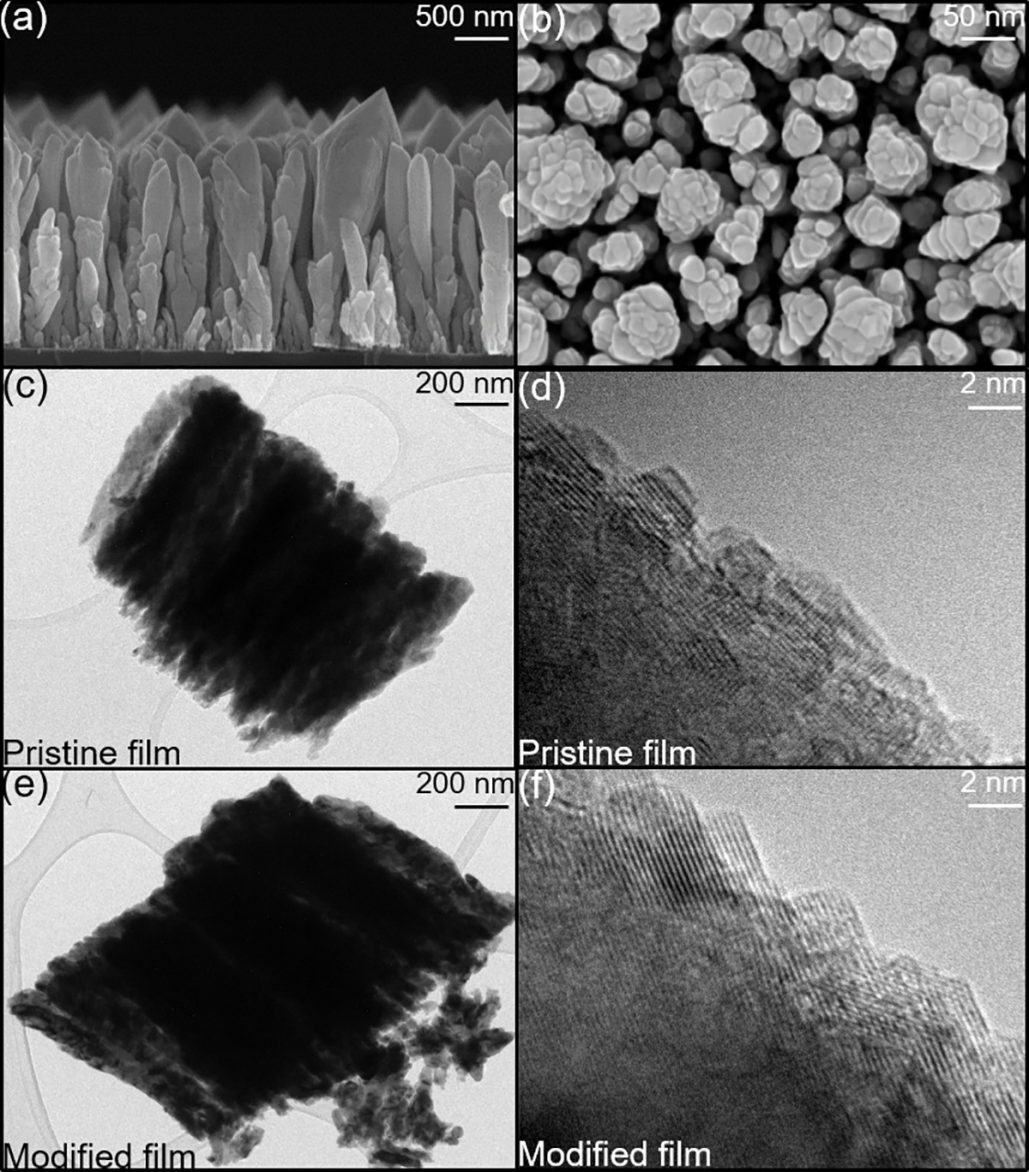
(a, b) SEM images of the pristine GLAD Ni film including cross-sectional (a) and top (b) views. (c-f) HRTEM images of pristine (c, d) and NH_3_ plasma treated (120 s) (e, f) Ni GLAD film.

Nanocolumnar Ni films were deposited on Si substrates by GLAD and treated with NH_3_ plasma for different lengths of time to yield N-functionalized Ni GLAD films. The morphologies of these Ni films were investigated by SEM (Fig 2A and 2B), and in all cases, a vertical columnar structure with large intercolumn distance was observed. No significant morphology changes were identified by SEM following plasma treatment (S1 Fig), indicating that the plasma processing does not strongly affect the film morphology and associated surface area. The nanocolumnar Ni films were approximately 600 nm thick with intercolumnar spacing of 90–120 nm. As will be relevant below, these intercolumnar distances are significantly larger than all chemical reactants and should allow diffusion into the interior of the GLAD structures.

The pristine and plasma-modified Ni films were also analyzed by high-resolution transmission electron microscopy (HRTEM, Fig 2C–2F), and little structural variation was noted following plasma processing. Interplanar spacings of ~0.20 nm were measured for both pristine and plasma-modified films, which is close to the accepted value for (111) planes in face-centered cubic (FCC) Ni crystallites. This suggests that both films include FCC Ni crystallites, but due to the nature of this analysis technique, it does not necessarily indicate that this is the only–or even the most prevalent–crystal phase present.

**Fig 3:**
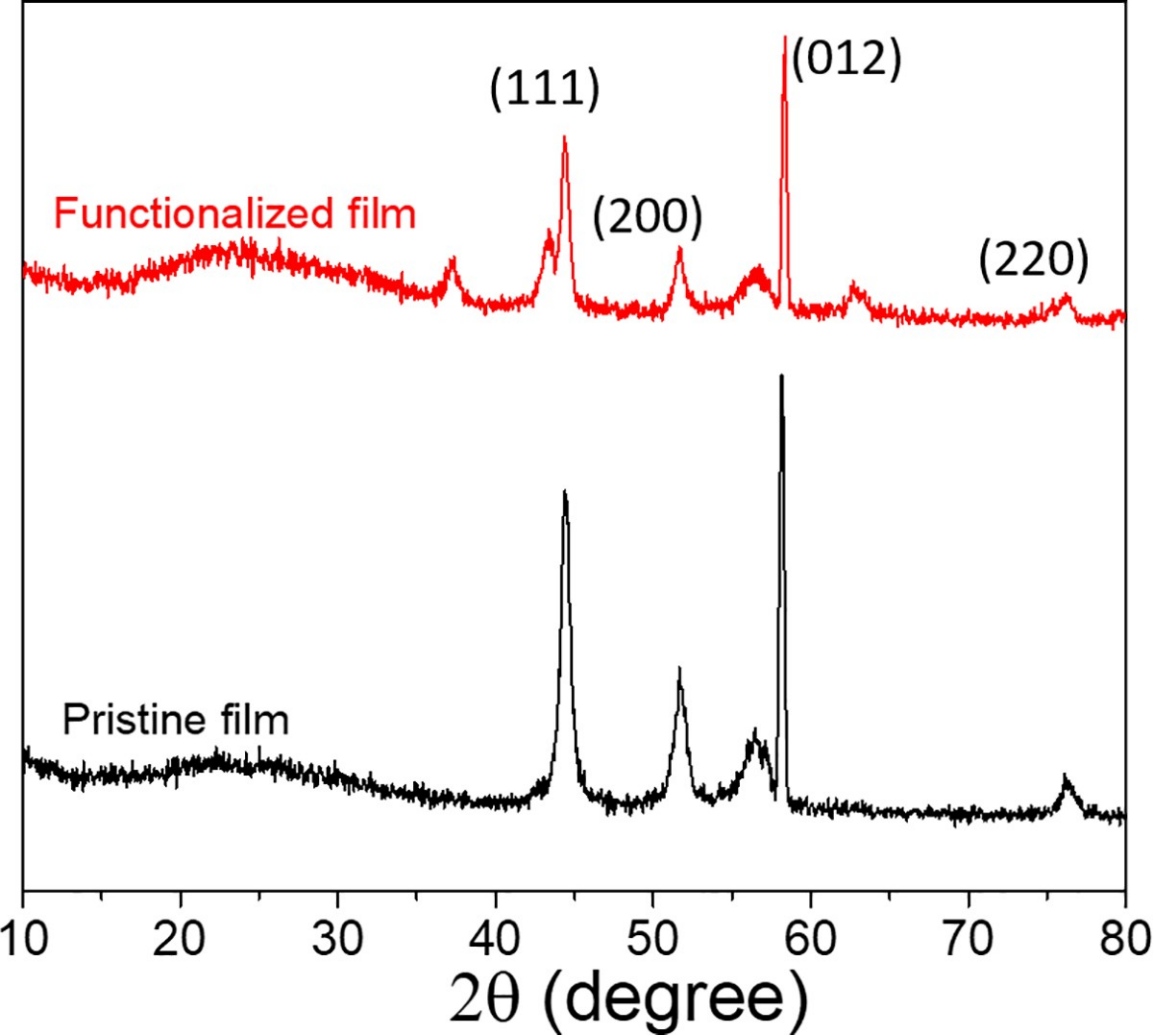
**XRD spectra for (black) pristine Ni film and (red) N-functionalized Ni film.** The N-functionalized film was treated for 120 s in NH_3_ plasma.

The crystal phase structure of the Ni films was investigated by x-ray diffractometry (XRD), and the spectra for both pristine and plasma-treated Ni films are shown in Fig 3. The spectrum for the pristine Ni film generally suggests a non-uniform, semi-crystalline structure with mixed phases. Diffraction peaks at 44.4°, 51.6° and 76.2° correspond to the (111), (200) and (220) planes, respectively, for FCC Ni (JCPDS card no. 00-001-1258) [26], while the peak at 58.1° suggests the (012) plane of HCP Ni (JCPDS card no. 45–1027) [27], and the peak at 56.7° suggests the (202) plane for Ni_2_O_3_ (JCPDS card no. 14–0481) [28]. All of these peaks are retained in the diffractogram for the NH_3_ plasma-treated film, and three additional peaks attributed to NiO (111), (200) and (220) planes are now observed at 37.3°, 43.4° and 62.9° (JCPDS card no. 22–1189) [29]. This new crystal phase likely arises as a result of the high temperature processing, but it is clear from the overall XRD pattern that the entire structure has not transitioned to this new phase. Overall, this XRD data suggests complex, non-equilibrium structures incorporating both Ni and NiO_x_, and this is true for both the pristine and plasma-modified films.

In addition, we note that each of the peaks originally observed in the pristine Ni film has shifted to slightly smaller 2θ positions (by approximately 0.3°), potentially indicating lattice strain from O or N atom insertion [30].

**Fig 4:**
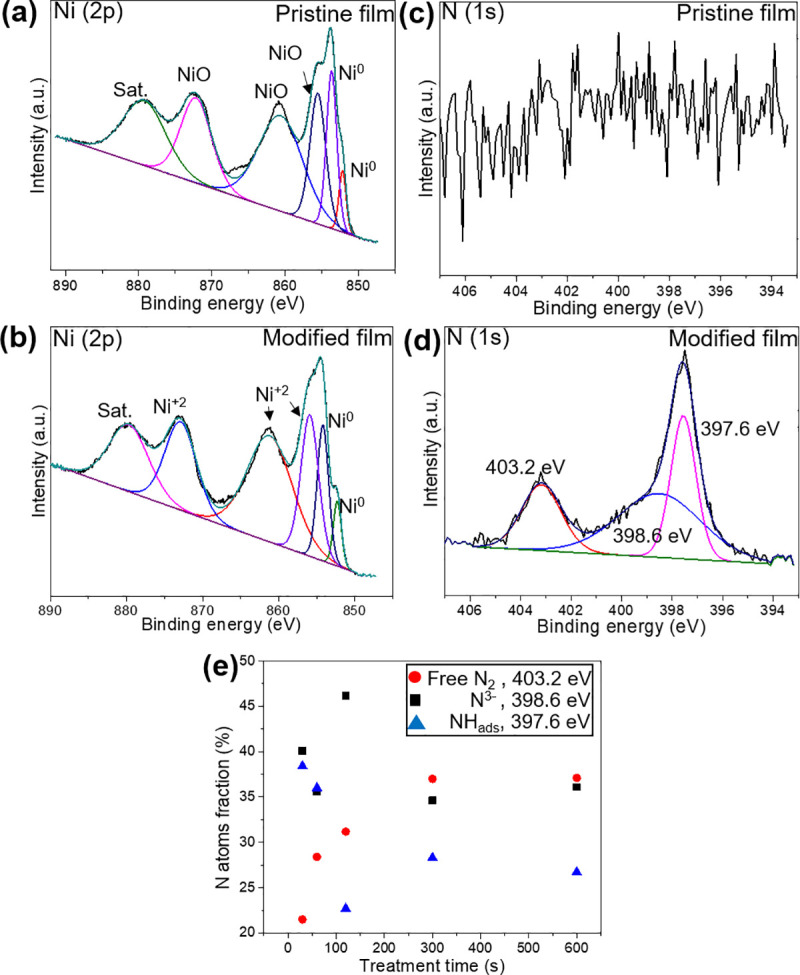
XPS spectra for pristine (a, c) and nitrogen-functionalized (b, d) Ni films in the binding energy regions associated with Ni(2p) (a, b) and N(1s) (c, d). In (b, d), the GLAD Ni films have been treated with NH_3_ plasma for 120 s. In (e), the fraction of nitrogen atoms present as adsorbed ammonia (NH_ads_), N^3−^ ions (N^3-^), and molecular nitrogen (N_2_), are plotted versus NH_3_ plasma treatment time.

The surface chemical composition and elemental valence states of the pristine and plasma-modified Ni films were also investigated by X-ray photoelectron spectroscopy (XPS), and the results are shown in Fig 4. Pronounced peaks for Ni are present for both pristine and plasma-treated films (Fig 4A and 4B), but N could only be distinguished in the plasma-treated samples (Fig 4D). In Fig 4A, peaks located at 852.1 eV and 853.6 eV indicate that metallic Ni^0^ is present [31], and Ni(2p) peaks at 855.5eV, 860.6 eV, and 872.1 eV (with its satellite peak present at 879.2 eV) also confirm the presence of Ni^2+^ on the pristine Ni films, likely in the form of NiO [32]. The same peaks are also observed for the Ni films after exposure to NH_3_ plasma (Fig 4B), however, the peaks have shifted to slightly higher binding energies. Ni^0^ is observed at 852.3 eV and 854.2 eV, and Ni^2+^ at 855.9 eV, 861.1 eV and 872.8 eV (with its satellite peak at 879.6 eV) [33]. This small shift may indicate that a fraction of the bound oxygen has been replaced with more electronegative nitrogen species.

Additional evidence for this effect is found in the N(1s) spectra in Fig 4C and 4D. No N(1s) signal was found for the pristine Ni film (Fig 4C), but after NH_3_ plasma treatment, strong peaks located at 397.6 eV, 398.6 eV, and 403.2 eV are visible in the XPS spectrum (Fig 4D), which can be assigned to adsorbed ammonia in the forms of NH (NH_ads_), N^3−^ ions (N^3-^), and molecular nitrogen (N_2_), respectively [34, 35]. Based on the N(1s) XPS spectra of the plasma-treated films (Fig 4E), we found that the fraction of nitrogen atoms present as NH_ads_ was at a minimum for films treated for 120s, while the fraction of N^3-^ ions was at a maximum. As nitrogen has a more negative valence state than oxygen, the incorporation of N^3−^ leads to oxygen (O^2−^) displacement. Overall, these results suggest nitrogen doping within the Ni/NiO crystalline lattices [36, 37].

### Plasma assisted enhancement of catalysis performances

The peroxidase-like catalytic activity of N-functionalized and pristine Ni GLAD thin films was investigated by immersing the catalytic Ni films in TMB solutions (0.4 mM) for 15 minutes at room temperature and pH 5 in the presence of 470 mM H_2_O_2_, thereby oxidizing the TMB (and causing the solution to change from colorless to blue). The data supporting the choice of these conditions is provided in S2 Fig, and a brief description of the rationale follows. Both H_2_O_2_ concentration and contact time between the TMB solution and catalytic film play vital roles in the formation of oxTMB solutions [13], as reflected by the strong correlations between each of these parameters and the absorbance at 652 nm (S2A–S2C Fig). Notably, we did not observe considerable oxidation of TMB by either N-functionalized or pristine films in the absence of H_2_O_2_, indicating that the films do not exhibit oxidase-like activity. 470 mM H_2_O_2_ was fixed as per previously reported literature for similar TMB concentrations [33, 38], and as per the literature, excess H_2_O_2_ in the TMB+H_2_O_2_ reaction was avoided to prevent two-electron conversion of oxTMB into the yellow colored diimine form [35]. We fixed the contact time at 15 minutes as increasing the time further did not substantially increase the absorbance. Room temperature was chosen both for simplicity as well as producing the optimum absorbance [39], and pH 5 was chosen based on our previous work [13] and its physiological relevance to study peroxidase mimicking behavior [40]. It should also be noted that the reaction rates vary with the thickness and area of the GLAD Ni film (S3 Fig), so these values were kept consistent throughout the study, i.e. 0.7 cm^2^ film area with 600 nm Ni thickness.

**Fig 5:**
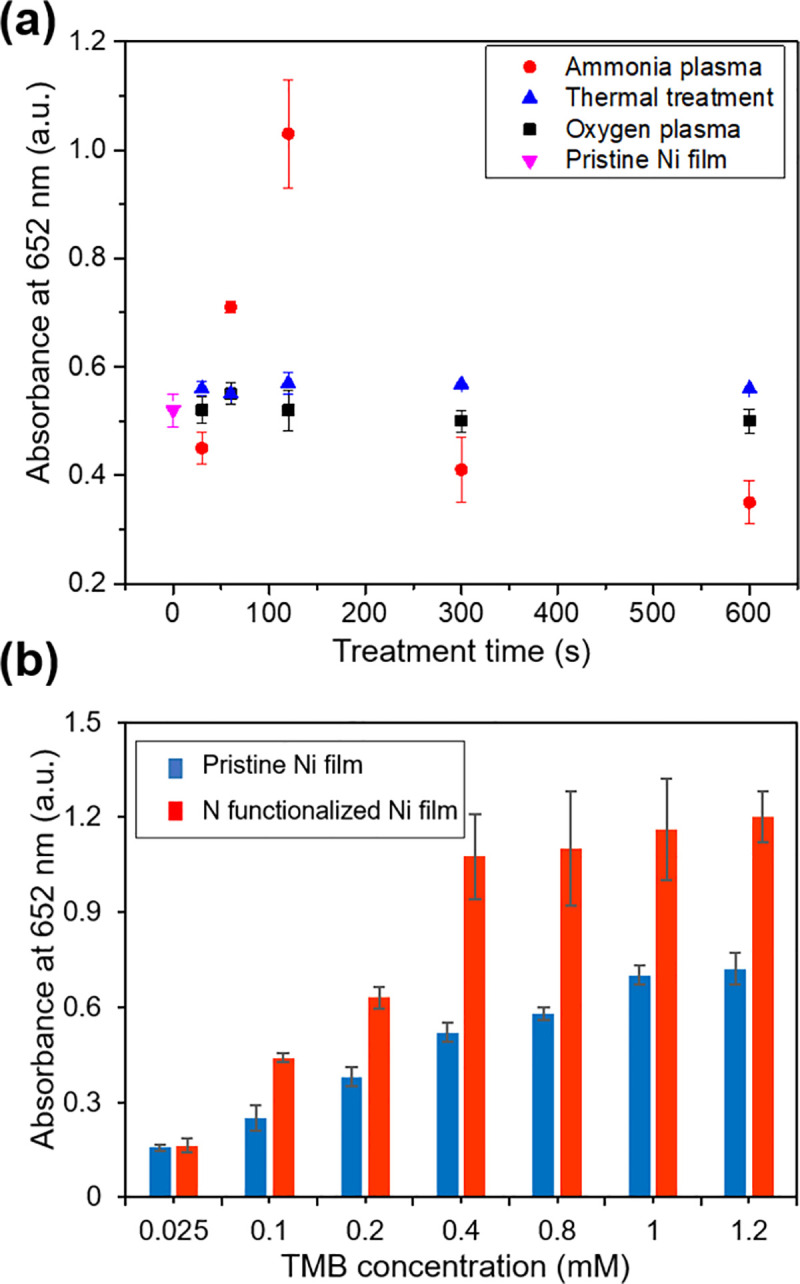
(a) Comparison of absorbance at 652 nm for equivalent TMB solutions oxidized by Ni GLAD films that were exposed to ammonia plasmas (red circles), oxygen plasmas (black squares) and plasma-free thermal treatments at 350°C (blue triangles) for various lengths of time. The original reaction solutions were always 525 μl (0.4 mM TMB and 470 mM H_2_O_2_), and the oxidation process was allowed to proceed for 15 minutes. (b) Comparison of absorbance at 652 nm for pristine Ni films and N-functionalized Ni films exposed for 120s to different concentrations of TMB.

Fig 5A shows that the absorbance at 652 nm increased to a maximum after 120 s of NH_3_ plasma treatment, then declined with additional exposure. The absorbance of solutions exposed to control films, prepared by exposing Ni GLAD films to either an oxygen plasma or to a high temperature (350°C), plasma free-environment (black squares and blue triangles, respectively, in Fig 5A), differed little from the absorbance of solutions exposed to pristine Ni films, suggesting that incorporation of nitrogen is responsible for the increased absorption. This behavior is consistent with previous work from Feng et al., who showed that a 2 min NH_3_ plasma treatment of MoS_2_ films led to 3-fold greater catalytic activity than pristine MoS_2_ [14].

The peroxidase-like performance of plasma treated films and pristine thin films were compared with varying concentrations of TMB. In its oxidized form, the chromogenic reagent TMB appears blue in solution (detectable at 652 nm by UV-Vis), and thus increasing the TMB concentration in the reaction mixture was expected to lead to greater absorbance at 652 nm. This effect was observed in Fig 5B. Lesser absorbance changes were observed beyond 0.4 mM, and thus 0.4 mM TMB became a standard throughout this research study.

In terms of mechanism, the catalytic surface of the nanozyme plays an important role in the oxidation of the substrate (the TMB) and the reduction of H_2_O_2_. Lone pair electrons from the adsorbed TMB are transferred to the catalyst [14, 41–44], and studies of metallic nanozymes have shown that an increase in electron density and conductivity occur during this process [45, 46]. Electrons are then transferred from the catalyst to peroxide, which is reduced to form water. In the literature, nitridation of a variety of metallic nanozymes has led to an enhancement in peroxidase-like activity [14, 41–44]. The enhanced catalytic activity of N-modified materials have been attributed to numerous factors: the strong affinity of N atoms for the TMB lone pairs [47], an increase in defect states (in MoS_2_ nanoflowers) [42], and increased wettability of the surface [14]. Comparing the contact angles for pristine and modified films (Table 1) is also revealing. Reduced contact angles were observed for plasma-treated films with respect to the pristine, as-deposited films (0 s plasma treatment), suggesting the incorporation of polar surface groups and possibly improving the ability of the redox species (i.e., TMB and H_2_O_2_) to access the catalytic surfaces. Additional factors mentioned above may also contribute to the enhanced catalytic activity of the N-modified films.

**Table 1:** Contact angles of water droplets on ammonia and oxygen plasma-treated Ni film for different treatment time.

Treatment time (s)	Contact angle (°)	
	O_2_ plasma	NH_3_ plasma
0	39.4	43.3
30	15.5	37.0
60	11.4	42.1
120	9.6	26.6
300	8.6	41.8
600	8.6	33.4

### Kinetic studies of the peroxidase-like reaction

**Fig 6:**
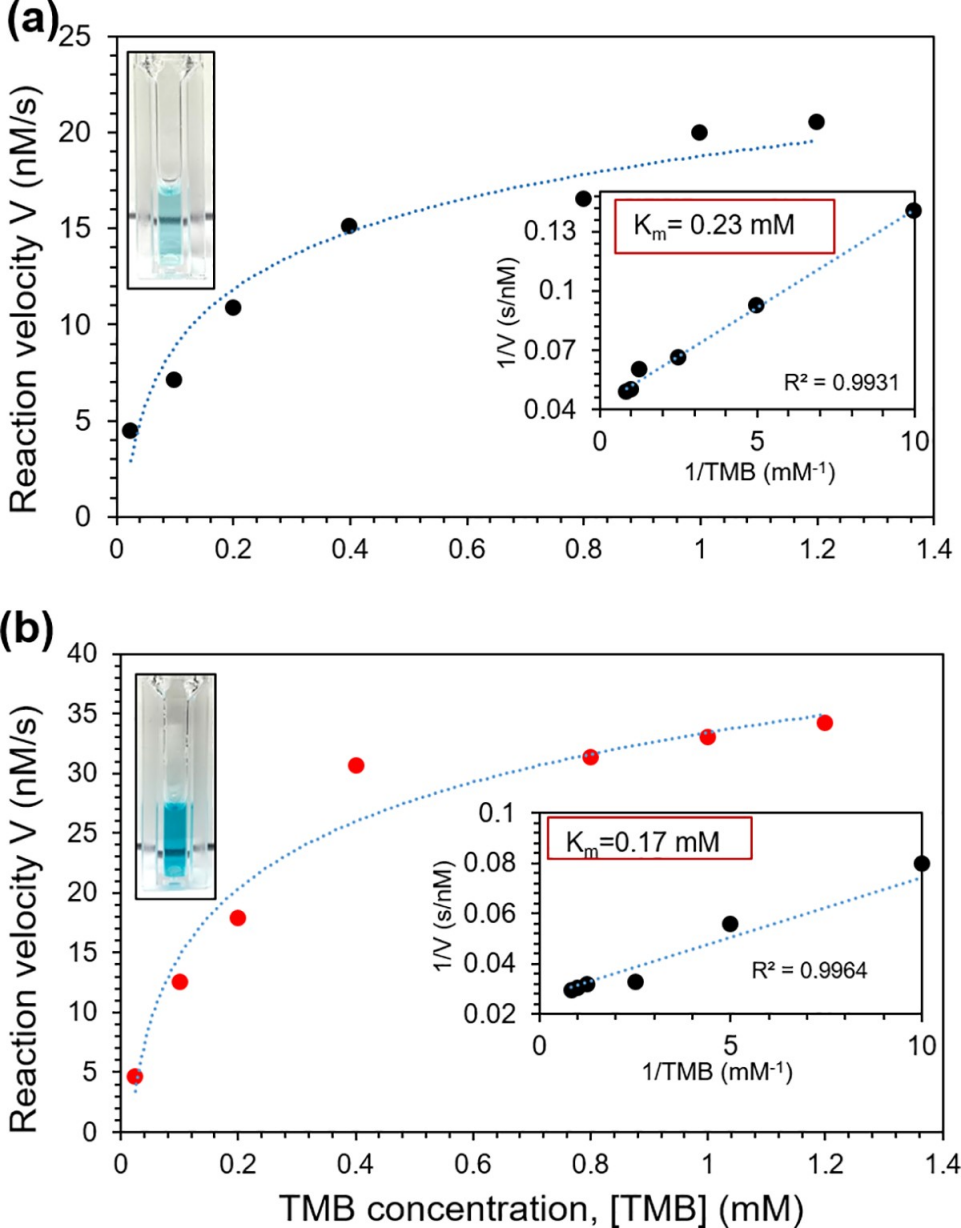
TMB conversion rate vs TMB concentration. (a) pristine Ni film, and (b) NH_3_ plasma modified Ni film for 120s.

Kinetic studies were carried out to compare the catalytic performances of N-functionalized and pristine Ni films using the TMB substrate, with the results shown in Fig 6. The kinetic properties of nanozymes vary with the concentration of substrate (reactant) present, as described by Michaelis-Menten kinetics. At low substrate concentrations the reaction rate (*V*) is linearly related to the substrate concentration [TMB], and the reaction exhibits first-order kinetics. At high concentrations, the rate of reaction (*V*_*max*_) is limited by the availability of active sites on the catalyst, and thus follows zero-order kinetics (i.e. *V* is independent of substrate concentration). The Michaelis-Menten constant *K*_*m*_ describes the substrate concentration at which the rate of reaction is half of *V*_*max*_, and is indicative of the affinity of the substrate for the catalyst. An effective catalyst exhibits a high *V*_*max*_ and low *K*_*m*_ value, indicating that the catalytic reaction can occur at a high rate over a useful range of substrate concentrations.

The Michaelis-Menten kinetic parameters were obtained by fitting the absorbance values for the catalytic reaction products versus time with varying concentrations of TMB. In Fig 6, the reaction rates for N-functionalized thin films generally exceed those for the pristine thin film, suggesting enhanced peroxidase-mimicking behavior. Accordingly, the kinetic parameters including the maximal reaction velocity (*V*_*max*_) and Michaelis-Menten coefficient (*K*_*m*_) for the N-functionalized film (3.7 ⊆ 10^−8^ M/s and 0.17 mM) are superior to those of the pristine film (2.3 ⊆ 10^−8^ M/s and 0.23 mM) showing that the catalytic properties have been enhanced. For comparison, the *K*_*m*_ and *V*_*max*_ values of a variety of nanozymes from the literature (evaluated using TMB) are shown in Table 2. Direct comparison of different materials is difficult as testing is done under different conditions, and enzymatic activity varies with both pH and temperature. Many of the reported values were collected around pH 3 and 37°C as enzymatic activity tends to be high under these conditions. In our studies, we selected a pH of 5 (so as to not damage the nanozyme film) and a temperature of 22°C (for simple operation). Varying these parameters will lead to different reaction kinetics. The surface-anchored GLAD film, which has the outstanding advantage of reusability, also leads to imperfect side-by-side comparisons with the solution-dispersed nanozymes. Nonetheless, our materials have competitive *V*_*max*_ values and lower *K*_*m*_ values as compared with other nanozymes. Notably, the *K*_*m*_ value for the N-functionalized film is considerably lower than all other literature reports for similar chromogenic TMB-H_2_O_2_ oxidation systems, showing that the N-functionalized thin film has a high tendency to bind to its TMB substrate, resulting in higher response.

**Table 2:** K_m_ values for different peroxidase-mimicking materials.

Catalyst	K_m_(mM)	V_max_ (10^−8^ M s^-1^)	Ref
MIL-53 (Fe)	1.08	3.12	[48]
MoS_2_ NPs	0.52	5.16	[49]
MoS_2_-Pt_74_Ag_26_	25.71	7.29	[50]
GO-Fe_3_O_4_	0.43	13.08	[51]
Hemin-graphene	5.10	4.5	[52]
Hemin	4.84	4.6	[52]
Au-NPs/graphene	0.14	7.1±0.1	[53]
N-doped graphene quantum dots	11.19	0.38	[54]
HRP	0.43	10	[9]
MoS_2_	0.82	1.16	[14]
Ni helical GLAD film	1.07	2.6	[13]
N-doped MoS_2_	0.79	1.79	[14]
Ni nanocolumnar GLAD film	0.23	2.3	This work
N-functionalized Ni nanocolumnar GLAD film	0.17	3.7	This work

### Application of GLAD-based nanozyme for continuous catalytic reaction

Various types of analysis techniques such as colorimetry, fluorescence, electrochemical sensing, chemiluminescence, and surface-enhanced Raman spectroscopy have been implemented in point-of-care diagnostic (POC) devices, including immunostrips and microfluidic devices [36, 55]. Some advances have been made for detection through strip-based tests, commonly known as lateral flow based assays. Lateral flow based assays are an advantageous diagnostic test configuration with features including less time consumption, easy operation, stability, low cost for POC devices, and smaller sample volumes [56], however, immobilizing enzymes on POC devices without compromising their function remains a challenge [57–59]. Self-driven flow over GLAD film-based nanozymes could be a unique fit for POC diagnostic platforms due to their excellent peroxidase-like activity, reusability (as shown in our previous work [13]), and high stability under a variety of storage conditions.

**Fig 7:**
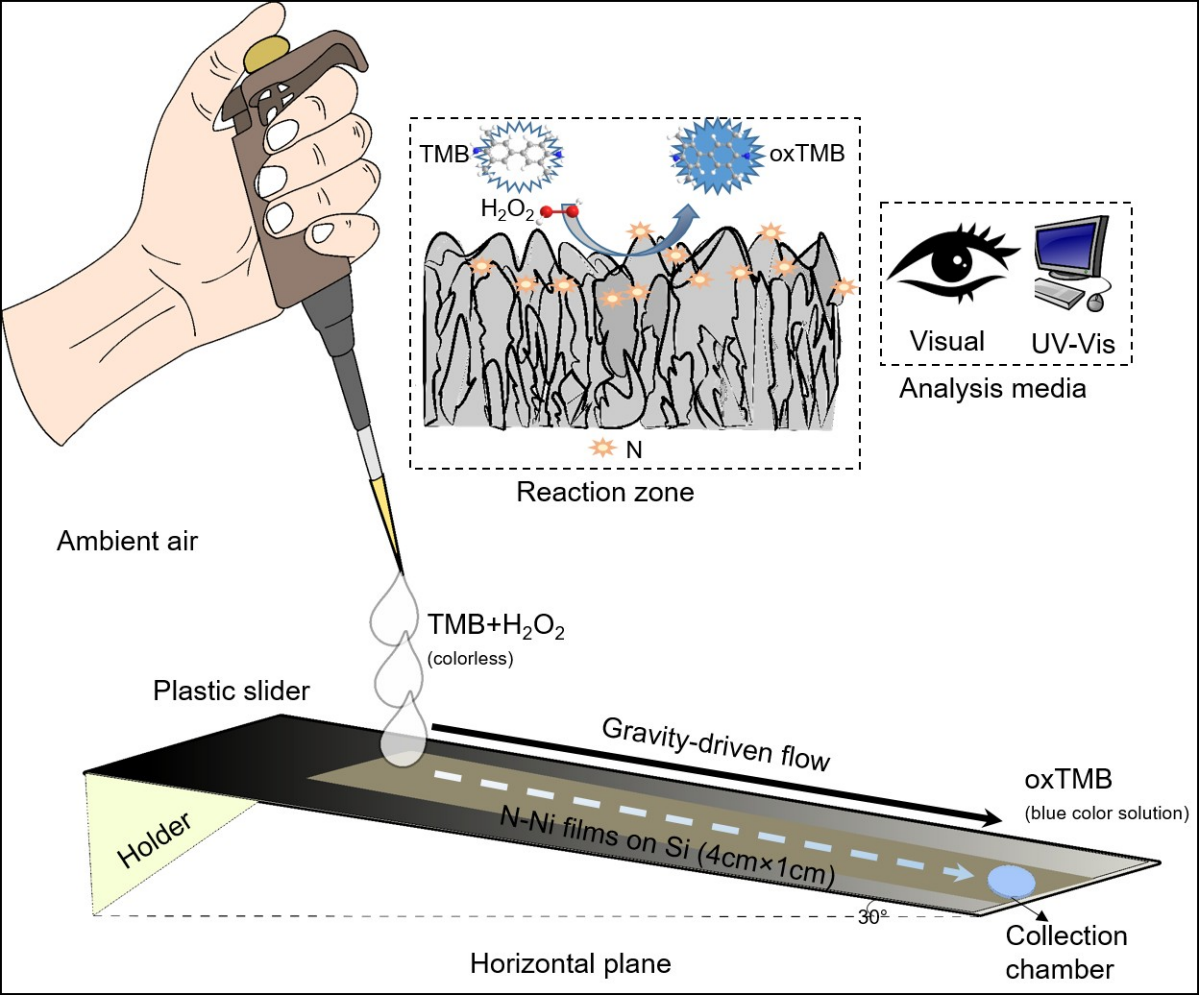
Schematic representation of gravity assisted flow device.

In Fig 7, we present a simple gravity-assisted device for continuous catalytic reaction on N functionalized GLAD films, and we adapt the device to function as a sensor for uric acid (UA), which is an important biomarker of human health conditions such as gout, arthritis, heart diseases, and kidney stones. As noted above, GLAD Ni catalysts facilitate the decomposition of hydrogen peroxide to generate oxygen species and form the blue-colored oxTMB from colorless TMB solution. In our previous work [13], when UA was added to the oxTMB solution, the blue color faded in proportion to UA concentration. Here, we adapted this system to demonstrate a gravity-driven device as a UA sensor by combining various concentrations of dilute UA with the TMB/H_2_O_2_ reagent mixture. We used 170 μl of 0.4 mM TMB, 170 μl of 470 mM H_2_O_2_, and 160 μl of UA solution (500 μl in total), and the combined solutions traveled across a catalytic GLAD Ni film because the substrate tilted at an angle of 30°. The flow was complete in ~3 minutes, and no external power source was required. As demonstrated in the S1 Movie (with 0 mM UA concentration), the colorless reactants became blue after traversing the reaction substrate. We found that this blue oxTMB solution could be wicked into cellulose paper or collected by pipette for additional analysis, and the initially colorless TMB solution (0.08 a.u.) reached a much stronger absorbance (0.51 a.u.) due to the ongoing oxidation reaction.

**Fig 8:**
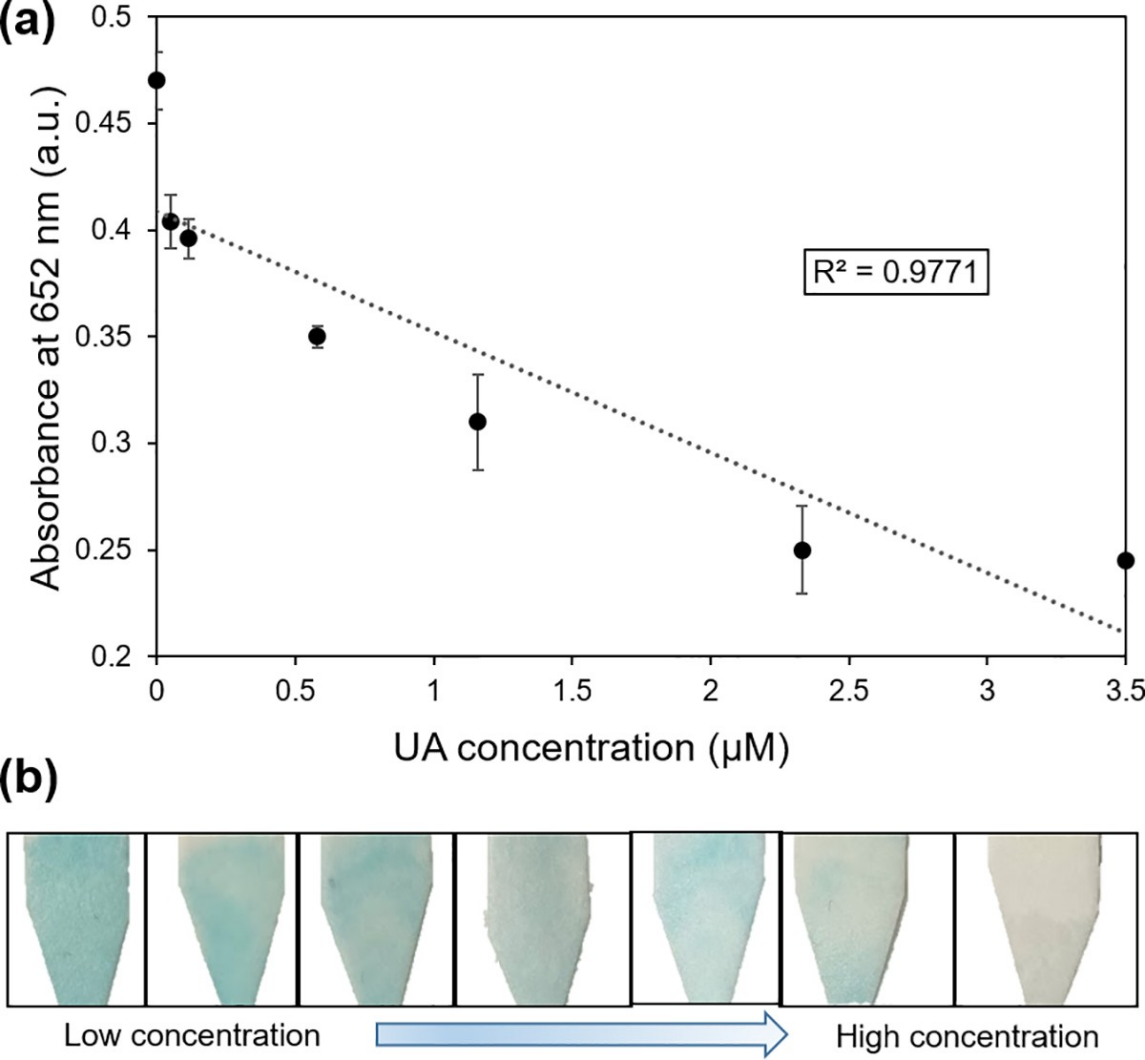
(a) Response of the gravity-driven device to variations in UA concentration from 0 μM to 3.5 μM. (b) Photographs show respective solutions collected on filter paper.

After the three component solution traverses the device, we expected higher concentrations of UA to lead to lower absorbance solutions collected at the outlet. In Fig 8, we provide the calibration data, and it can be noted that as the UA concentration increases from 0 μM to 3.5 μM, the solution absorbance decreases from 0.47 to 0.24 with an R^2^ value of 0.97. In this configuration, the limit of detection (LOD) that was achieved was 0.98 μM (calculated as 3σ/slope). No external power sources were required, and the solutions were all generated from the same plasma-treated Ni thin film, demonstrating reusability and opening the way toward potential application in colorimetric sensors.

## Conclusions

An effective, environmentally-friendly GLAD film-based biosensor was improved using a simple ammonia-plasma treatment process. The resulting surface-mounted nanozymes presented dramatically enhanced catalytic performance (with respect to untreated pristine counterparts) depending on the plasma treatment conditions. SEM, TEM, XRD, and XPS analysis results revealed that the plasma treatment introduced N-rich surface species, and greatly increased surface wettability was observed, allowing easier access for reactants. Although the exact catalysis mechanism may be further explored, the enhanced catalytic performance parameters demonstrated for a reusable nanozyme may open the door towards electrochemical and colorimetric sensors for environmental monitoring, food safety, and biomedical analysis. The demonstration of continuous catalytic activity (via gravity-driven flow across the film) represents one additional step toward these real-world applications, particularly for integration in flow-driven point of care devices.

## Materials and methods

### Reagents

3,3’,5,5’-Tetramethyl benzidine (TMB), hydrogen peroxide, isopropanol (IPA), dimethyl formamide (DMF), and phosphate citrate buffer (pH 5) were purchased from Sigma-Aldrich. Deionized water (0.055 μS) was used throughout this work. All chemicals were of analytical grade and were used as received without further purification.

### Preparation of N-functionalized thin films

Ni thin films were deposited on piranha cleaned silicon wafers by electron beam evaporation. The base pressure prior to deposition was <2×10^−6^ Torr, and the pressure during the deposition was roughly 1×10^−5^ Torr. The deposition angle between the incident vapor flux and the substrate normal was 80°, and during deposition, the substrate was rotated at a rate of one complete revolution for every 100 nm of film growth. Ammonia plasma treatment was performed in an instrument primarily intended for plasma-enhanced chemical vapor deposition: a Trion Orion PECVD. The films were exposed to a 220 W RF ammonia plasma at a temperature of 350°C. The ammonia flow rate was 20 sccm, and the operating pressure was kept at 400 mT.

To produce a reference sample, a plasma-free thermal treatment at 350°C was also performed in the Trion Orion PECVD. Ni GLAD films were inserted into the chamber, which was heated to 350°C and evacuated, however neither gas flows nor RF power were applied, so these Ni control films were thermally annealed under vacuum. A second reference sample was prepared by exposing a Ni GLAD film to an oxygen plasma treatment, executed in a Trion RIE at room temperature. A 100 W RF oxygen plasma was formed, where the oxygen flow rate was 98 sccm, and the operating pressure was 100 mT.

### Apparatus

High-resolution scanning electron microscopy (SEM) was performed using a Hitachi S5500, and high-resolution transmission electron microscopy (HRTEM) was performed using a Hitachi H-9500 instrument. X-ray photoelectron spectroscopy (XPS, Kratos AXXIS Ultra) was used to analyze the surface composition of the GLAD film. Samples were irradiated with a monochromatic Al Kα source (h*v* = 1486.71 eV), and the pressure of the analysis chamber was below 5×10^−10^ Torr during the elemental analysis. The phase compositions of the prepared materials were determined by X-ray powder diffractometry (Rigaku XRD Ultima IV). The diffraction patterns of samples were recorded in the range of 2θ = 10–80° using a Cu radiation source with fixed power (40 kV, 44 mA). Contact angles of purified water droplets on Ni GLAD films were monitored using a First Ten Angstroms contact angle goniometer (FTA-200).

### Measurements of peroxidase-like catalytic performances

Colorimetric investigations of the peroxidase-like catalytic performances were conducted using the chromogenic TMB-H_2_O_2_ reaction in phosphate citrate buffer (pH5). For the basic reaction, 0.7±0.02 cm^2^ of Ni film (S4 Fig) was immersed in 525 μl of solution containing 0.4 mM TMB and 470 mM H_2_O_2_. Contact was maintained for 15 minutes, then the film was removed, and the absorbance spectra recorded. For optimization of the catalytic reaction conditions, the parameters were varied as noted in the accompanying text with all other conditions fixed as above.

### Studies of Michaelis-Menten kinetics

The studies of the steady-state catalysis kinetics were performed at a fixed concentration of 470 mM H_2_O_2_ and varying concentrations of TMB. The absorbance values were recorded at 652 nm, and the kinetic catalysis parameters were obtained by linear fitting of the Lineweaver-Burk double-reciprocal plot. The Michaelis-Menten constant (K_m_) and the maximal reaction velocity (V_max_) were extracted from:

$$\frac{1}{V}=\frac{K_{m}}{V_{\max}[\mathrm{TMB}]}+\frac{1}{V_{\max}}$$

where, V is the initial reaction velocity, and [TMB] refers to the substrate concentration.

## Supporting information

S1 Fig SEM images of pristine and N-functionalized Ni films. (TIF)

S2 Fig Optimal reaction conditions for peroxidase-like activity. (TIF)

S3 Fig Effect of thickness and area of Ni films on absorbance. (TIF)

S4 Fig Photograph of Ni GLAD film. (TIF)

S1 Movie Gravity-driven Ni nanozyme for continuous catalytic reaction. (MP4)

S1 Data Data underlying the findings in the article. (ZIP)

S1 Graphical abstract (TIF)
