# Supplementary figures and images for: High surface area nitrogen-functionalized Ni nanozymes for efficient peroxidase-like catalytic activity
Source: PLoS One. 2021 Oct 12;16(10):e0257777. doi: 10.1371/journal.pone.0257777 (PMC8509884; doi:10.1371/journal.pone.0257777)

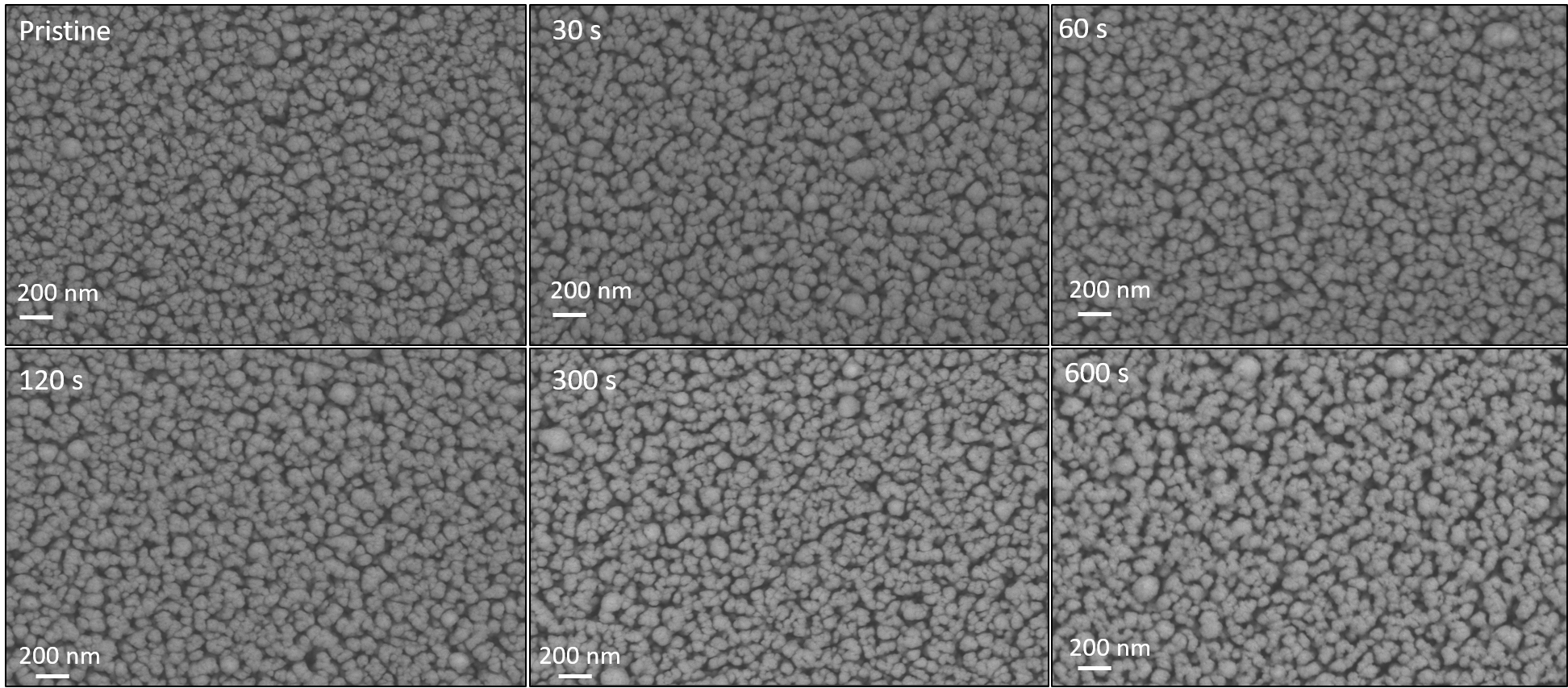

Supplement: S1 Fig — (TIF) [file pone.0257777.s001.tif]

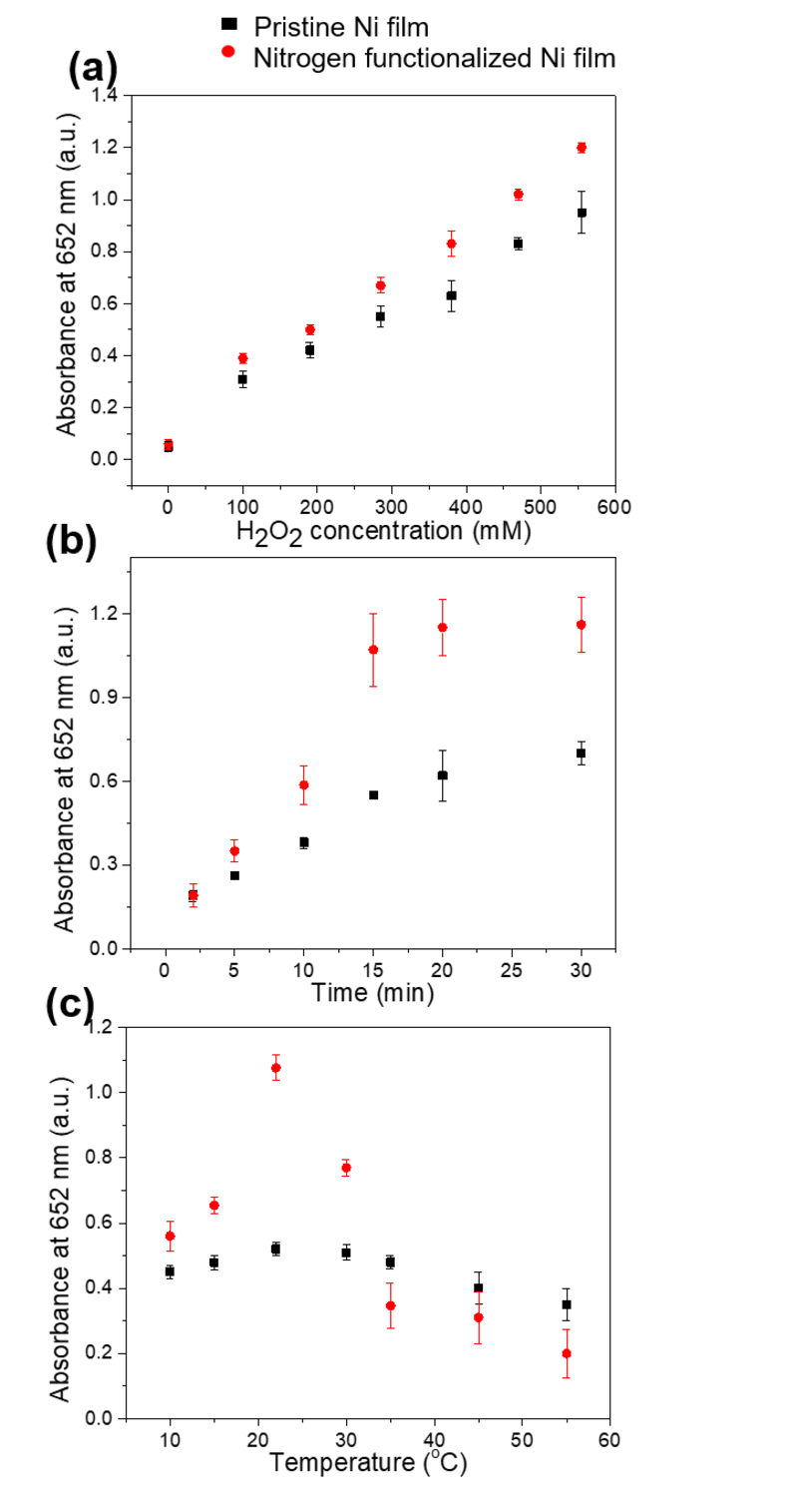

Supplement: S2 Fig — (TIF) [file pone.0257777.s002.tif]

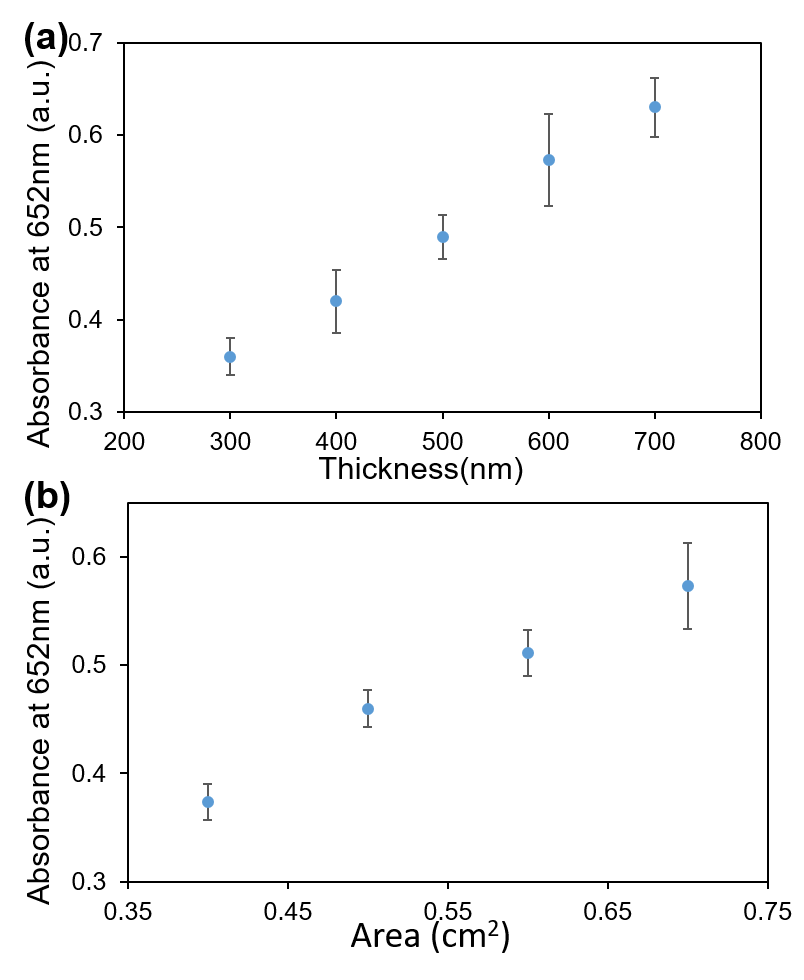

Supplement: S3 Fig — (TIF) [file pone.0257777.s003.tif]

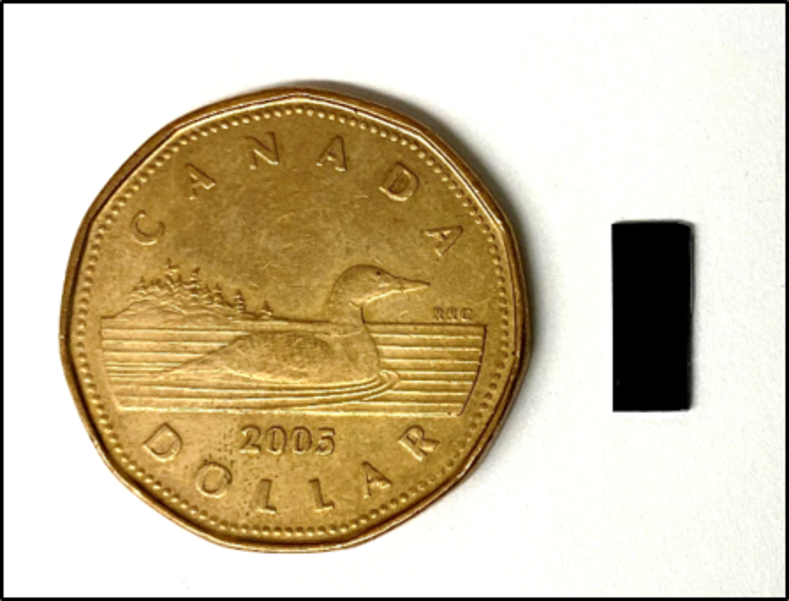

Supplement: S4 Fig — (TIF) [file pone.0257777.s004.tif]

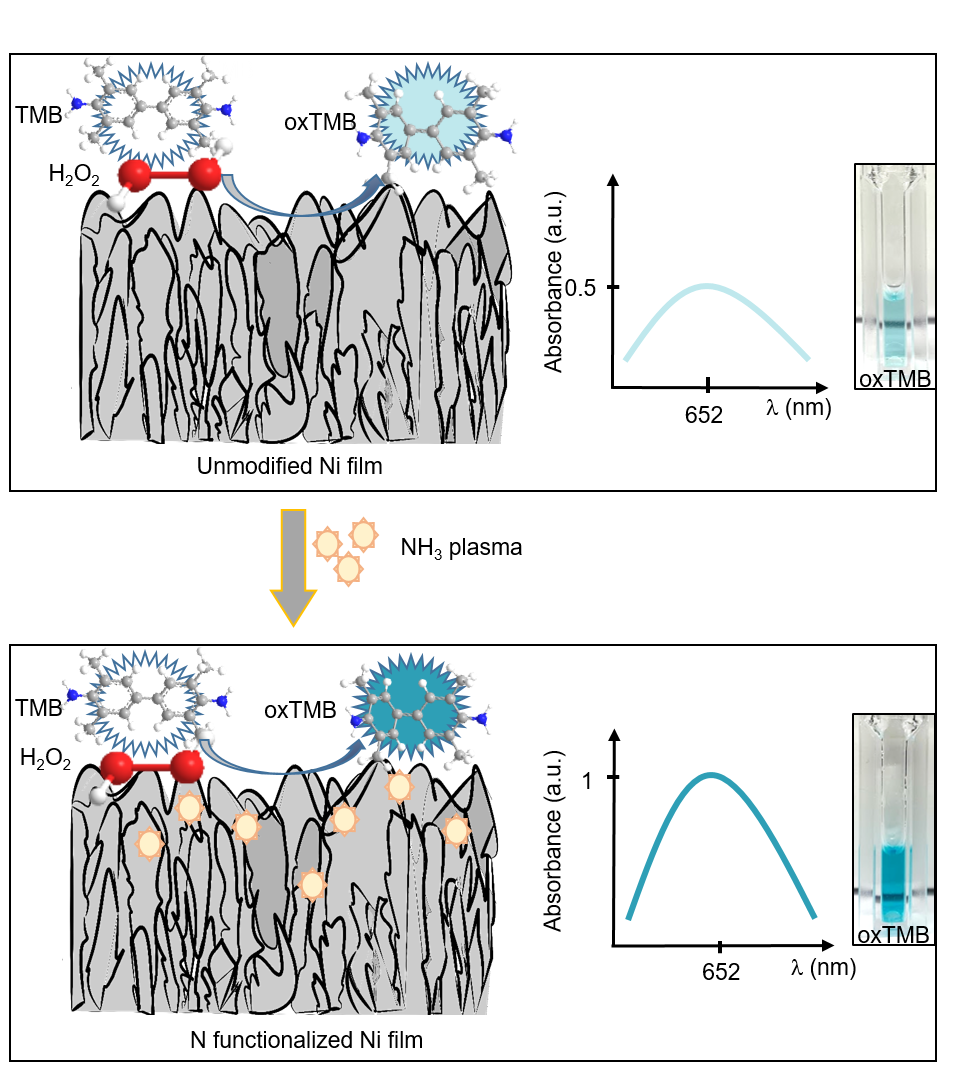

Supplement: S1 Graphical abstract — (TIF) [file pone.0257777.s007.tif]
